# A Case of Concurrent Gastric and Pancreatic Plasmacytomas in a Patient With Multiple Myeloma: An Extremely Rare Entity

**DOI:** 10.1177/2324709618777003

**Published:** 2018-05-24

**Authors:** Tagore Sunkara, Santosh R. Sharma, Andrew Ofosu, Vinaya Gaduputi, Madhavi Reddy, Ghulamullah Shahzad

**Affiliations:** 1 The Brooklyn Hospital Center, Clinical Affiliate of the Mount Sinai Hospital, Brooklyn, NY, USA; 2 SBH Health System, Bronx, NY, USA

**Keywords:** multiple myeloma, extramedullary multiple myeloma, gastric plasmacytoma, pancreatic plasmacytoma

## Abstract

Multiple myeloma (MM), a plasma cell tumor, is primarily a disease of the bone marrow. Extramedullary plasmacytoma, also a plasma cell tumor, is very rare in the gastrointestinal tract and the pancreas, and only a handful cases have been documented till now. Gastric and pancreatic plasmacytomas are usually seen in elderly patients; however, cases in patients as young as 32 years of age have been reported. Commonly, patients with gastric plasmacytoma present with nonspecific symptoms like epigastric pain, abdominal fullness, anorexia, and weight loss, or serious conditions like massive upper gastrointestinal bleeding and gastric outlet obstruction. Patients with pancreatic plasmacytoma commonly present with obstructive jaundice. In this article, we present the case of a 79-year-old man with a history of MM for 3 years, diagnosed with gastric and pancreatic masses, which turned out to be plasmacytomas. To our knowledge, simultaneous occurrence of gastric and pancreatic plasmacytomas is extremely uncommon with less than 5 cases reported in the literature. We also compiled all the individual cases of gastric and pancreatic MM that have been reported in literature till now.

## Introduction

Multiple myeloma (MM) is primarily the disease of the bone marrow characterized by abnormal proliferation of plasma cells.^1^ It is referred to as plasmacytoma when the lesions are sporadic and do not meet the systemic criteria for MM.^2^ Most commonly, plasmacytomas arises in the bone; however, it can be seen anywhere in the body.^2^ Extramedullary involvement of MM is very rare and most commonly seen in the respiratory tract, and less than 5% of the cases are seen in the gastrointestinal (GI) tract.^3^ We present the case of 79-year-old man who after being treated for MM was found to have a gastric mass and a pancreatic mass, which were diagnosed as plasmacytomas.

## Case Report

**Figure 1.**
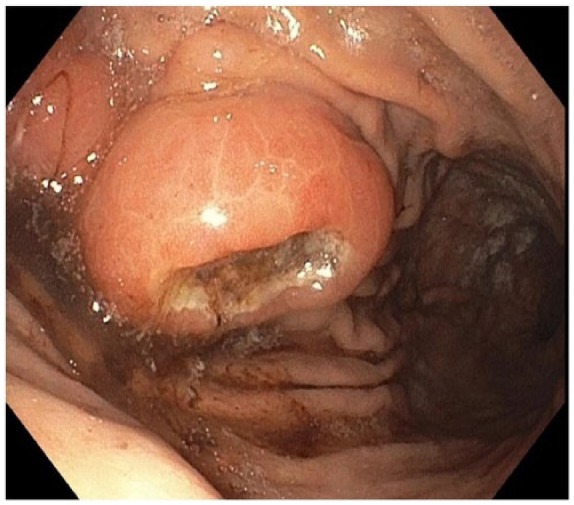
Esophagogastroduodenoscopy revealed a large polypoid mass with central ulceration in the gastric cardia.

**Figure 2.**
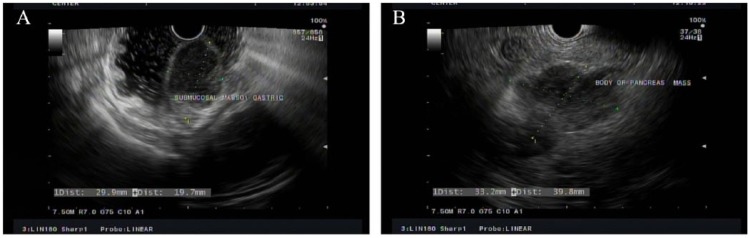
(A) Endoscopic ultrasound (EUS) showing the gastric mass arising from the submucosal gastric layer, measured 29.9 × 19.7 mm in diameter. (B) EUS showing an irregular, well-defined, and heterogeneous solid mass with cystic components in the body of the pancreas measured 33.2 × 39.8 mm in diameter.

**Figure 3.**
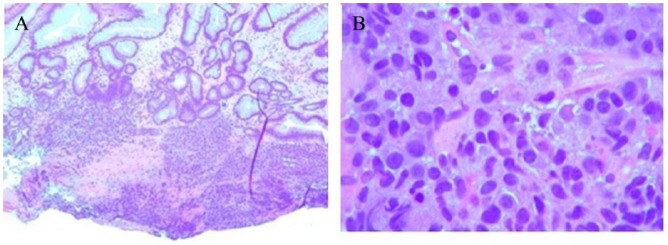
Hematoxylin and eosin staining. (A and B) Histology of the gastric biopsy showing the presence of abundant atypical cells, which had eccentrically placed large nuclei with occasional multinucleation and with few mitotic figures (magnification, ×40 and ×100, respectively).

**Figure 4.**
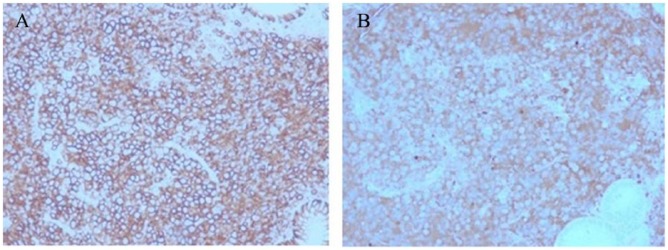
(A) On immunohistochemistry (IHC), the cells showing clusters of monoclonal plasma cells, stain positive for CD138 (magnification, ×100). (B) On IHC, the cells showing clusters of monoclonal plasma cells, stain positive for Kappa, which was consistent with plasma cell neoplasm (magnification, ×100).

A 79-year-old man with medical history of hypertension, diabetes mellitus type 2, and MM (phenotype IgA), which was treated with 4 cycles of lenalidomide plus dexamethasone 3 years prior to this presentation and in remission since then, was referred to the gastroenterology clinic for persistent epigastric pain and nausea. Physical examination was significant for epigastric tenderness on palpation, but otherwise unremarkable. His laboratory test values including liver function tests were within normal limits. The patient underwent esophagogastroduodenoscopy, which revealed a large polypoid mass with central ulceration in the gastric cardia (Figure 1). Subsequently, an endoscopic ultrasound (EUS) was performed to further evaluate the mass. On EUS, the gastric mass was seen arising from the submucosal gastric layer, measured 29.9 × 19.7 mm in diameter (Figure 2A). In addition, the EUS also revealed an irregular, well-defined, and heterogeneous solid mass with cystic components in the body of the pancreas (Figure 2B). It measured 33.2 × 39.8 mm on its long axis and 39.2 × 33.2 mm on it short axis. The mass was invading the splenic vein abutting the portal vein. There was no invasion of major vessels like hepatic artery, celiac artery, superior mesenteric artery, and superior mesenteric vein. The liver and the kidneys appeared normal. Multiple fine needle aspiration biopsies were taken from both the gastric and pancreatic masses using a 22G cook needle. The gastric biopsy was reported as showing the presence of abundant atypical cells, which had eccentrically placed large nuclei with occasional multinucleation and with few mitotic figures (Figure 3A and B). Immunohistochemically, the cells showed clusters of Kappa-restricted monoclonal plasma cells, positive for CD138 (Figure 4A) and κ (Figure 4B), which was consistent with plasma cell neoplasm. It was negative for Lambda, CK7, CK20, CDX2, synaptophysin, chromogranin, TTF1, CD68, melan-A, S-100, and CD45. Similarly, the biopsy from the pancreatic mass showed atypical cells suggestive for plasma cell neoplasm. The patient was diagnosed with gastric and pancreatic plasmacytomas and was referred to hematology-oncology for possible treatment options. Serum immunofixation was done, and it revealed 2 monoclonal bands present in the gamma region, both demonstrating IgA/Kappa identity. Protein electrophoresis revealed albumin (%) of 35.9%, which is low; gamma globulin (%) of 39.9%, which is elevated, and M-protein (%) of 35.6%, which is elevated. The total M-protein level on protein electrophoresis was 3.3 g/dL, which is elevated. A skeletal survey was performed to assess other sites for the disease. Skeletal survey showed a solitary radiolucent lesion in the left parietal lobe. Patient is started on 4 mg intravenous zoledronic acid once a month. The patient is currently on pomalidomide 2 mg for 21 days/month and 7 days break/month. The patient is clinically improving with guarded prognosis.

## Discussion

Multiple myeloma is a malignancy of the hematopoietic system characterized by an abnormal proliferation of plasma cells with overproduction of immunoglobulins in the bone marrow.^1^ Extraskeletal MM in the GI tract is rare,^4^ and the most common site is the small bowel followed by the stomach, colon, and esophagus. Involvement of the pancreas is even rarer, and only a few cases have been reported. Less than 5 cases of concurrent gastric and pancreatic involvement by MM have been reported in the literature. We compiled all the cases of gastric plasmacytoma (Table 1) and pancreatic plasmacytoma (Table 2) that have been reported in the literature.

**Table 1.** Reported Gastric Multiple Myeloma Cases in the Literature.

Study	Age/Sex	Number of Cases	Concomitant Involvement of Pancreas	Clinical Manifestation
Talamo et al^4^	Not documented	4	Yes/2 cases	1 case of nausea/vomiting with obstructive jaundice 1 case of upper GI bleeding 1 case of gastric outlet obstruction/ascites/MM cells found in ascetic fluid 1 case of an incidental finding
Griffiths et al^5^	68/Male	1	No	Recurrent bouts of hematemesis and melena
Goeggel-Lamping and Kahn^6^	71/Female	1	Yes	Diffuse abdominal pain, nausea, vomiting, intermittent diarrhea, and constipation
Sakai et al^7^	37/Male	1	No	No preceding history of multiple myeloma
Kusano^8^	64/Male	1	No	Has history of gastric cancer and multiple myeloma
Yasar et al^9^	69/Male	1	No	Massive upper GI bleeding
Sousos^10^	64/Male	1	No	Epigastric pain
Katzenberger^11^	79/?	1	No	Intractable vomiting
Elbaum et al^12^	68/Female	1	No	Crampy abdominal pain, fatigue, weight loss
Wang et al^13^	52/Female	1	No	Upper GI bleeding
Doberauer et al^14^	72/Male	1	No	Anemia and B_12_ deficiency
Kwak et al^15^	61/Male	1	No	Epigastric pain and weight loss
Birjawi et al^16^	50/Male	1	Yes	Unknown
Lu et al^17^	52/Female	1	No	Epigastric pain, anorexia, and fullness of abdomen
Kazama et al^18^	49/Female	1	Yes	Painless jaundice, pruritus, and hematuria

Abbreviations: GI, gastrointestinal; MM, multiple myeloma.

**Table 2.** Reported Pancreatic Multiple Myeloma Cases in the Literature.

Study	Age/Sex	Number of Cases	Concomitant Involvement of Stomach	Clinical Presentation
Talamo et al^4^	Not documented	5	Yes/2 cases	2 cases of jaundice due to obstruction of CBD 1 case of abdominal pain; nausea and vomiting 1 case of incidental finding in autopsy
Goeggel-Lamping and Kahn^6^	69/Female	1	Yes	Diffuse abdominal pain, nausea, vomiting, intermittent diarrhea, and constipation
Sciancalepore et al^19^	62/Male	1	No	Obstructive jaundice
Utsumi et al^20^	83/Male	1	No	Obstructive jaundice
Smith et al^21^	66/Male	1	No	Elevated transaminases and obstructive jaundice
Leake et al^22^	46/Male	1	No	Obstructive jaundice
Annibali et al^23^	45/Female	1	No	Obstructive jaundice
Birjawi et al^16^	64/Male 66/Male 50/Male	3	Yes/1 case	Unknown
Kazama et al^18^	49/Female	1	Yes	Painless jaundice, pruritus, and hematuria
Balliu et al^24^	32/Female	1	No	Jaundice
Fischer et al^25^	58/Male	1	No	Obstructive jaundice
Senzaki et al^26^	41/Male	1	No	Was diagnosed by autopsy
Mitchell and Hill^27^	Unknown	1	Unknown	Jaundice
Bell et al^28^	88/Male	1	No	Obstructive jaundice
Matsubayashi et al^29^	51/Male	1	No	Obstructive jaundice
Simon et al^30^	77/Female	1	No	Obstructive jaundice

Abbreviation: CBD, common bile duct.

From Tables 1 and 2, we can see that most of the patients are more than 60 years old; however, cases as young as 37 years of age for gastric MM and 32 years of age for pancreatic plasmacytoma have also been reported. Most of the patients with gastric plasmacytoma presented with expected symptoms of epigastric pain, anorexia, fullness of the abdomen, and weight loss like our case. Nonetheless, patients also presented with serious complications like massive upper GI bleeding and gastric outlet obstruction.^4,5,9,13^ There was a case where gastric plasmacytoma was diagnosed on a patient who presented with anemia and vitamin B_12_ deficiency.^14^ In contrast, there was relative uniformity in presentation in patients with pancreatic involvement. Most patients presented with features of obstructive jaundice, and one case was diagnosed during autopsy.^4,18-30^

Involvement of both the stomach and pancreas is very rare. When both organs had been involved, patients had features of both abdominal pain and jaundice. Few patients where both pancreas and stomach were involved had distant metastasis to other organs, and patients have limited survival. In contrast, our patient had only the involvement of the stomach and the pancreas without evidence of metastasis to other organs, and his symptoms were only abdominal pain without jaundice.

Almost half of the patients were diagnosed with MM after gastric plasmacytoma was diagnosed, while the remainder had been diagnosed with MM earlier ranging from a few months to up to 3 years. In case of pancreatic plasmacytoma, only 3 cases were newly diagnosed, and they had longer years of survival with MM before diagnosis of pancreatic plasmacytoma up to 13 years. Our patient was diagnosed with MM 3 years prior to diagnosis of gastric and pancreatic plasmacytoma.

Most of the cases of gastric and pancreatic plasmacytomas have been described as primary extramedullary plasmacytomas without the presence of systemic disease. However, finding a gastric or pancreatic plasmacytoma is an indication to rule out MM and surveillance of lesions elsewhere in the body.^3^ Treatment of choice for small lesions that can be completely resected is surgery and the role of radiotherapy is not well established. However, for lesions that cannot be completely resected, radiation therapy alone with a dose of 40 to 50 gyri over a 4-week period is recommended.^31,32^ There are no current recommendations to treat solitary plasmacytoma with chemotherapy.^31,32^ However, the presence of a solitary plasmacytoma anywhere in the body warrants to look for extensive disease (MM), which need to be treated with chemotherapeutic agents (like bortezomib or lenalidomide), or hematopoietic cell transplantation, or combination of both based on different patient factors.
